# Working with entrustable professional activities in clinical education in undergraduate medical education: a scoping review

**DOI:** 10.1186/s12909-021-02608-9

**Published:** 2021-03-19

**Authors:** Severin Pinilla, Eric Lenouvel, Andrea Cantisani, Stefan Klöppel, Werner Strik, Sören Huwendiek, Christoph Nissen

**Affiliations:** 1 University Hospital of Old Age Psychiatry and Psychotherapy, University of Bern, Bern, Switzerland; 2 Department for Assessment and Evaluation, Institute for Medical Education, University of Bern, Bern, Switzerland; 3 University Hospital of Psychiatry and Psychotherapy, University of Bern, Bern, Switzerland

**Keywords:** Entrustable professional activities, Undergraduate medical education, Clinical education, Scoping review

## Abstract

**Background:**

Entrustable professional activities (EPAs) are increasingly used in undergraduate medical education (UME). We conducted a scoping review to summarize the evidence for the use of EPAs in clinical rotations in UME.

**Methods:**

We searched multiple databases for scoping reviews based on the PRISMA guidelines for articles reporting qualitative and quantitative research, as well as conceptual and curriculum development reports, on EPAs in UME clinical rotations.

**Results:**

We identified 3309 records by searching through multiple databases. After the removal of duplicates, 1858 reports were screened. A total of 36 articles were used for data extraction. Of these, 47% reported on EPA and EPA-based curriculum development for clerkships, 50% reported on implementation strategies, and 53% reported on assessment methods and tools used in clerkships. Validity frameworks for developing EPAs in the context of clerkships were inconsistent. Several specialties reported feasible implementation strategies for EPA-based clerkship curricula, however, these required additional faculty time and resources. Limited exposure to clinical activities was identified as a barrier to relevant learning experiences. Educators used nationally defined, or specialty-specific EPAs, and a range of entrustability and supervision scales. We found only one study that used an empirical research approach for EPA assessment. One article reported on the earlier advancement of trainees from UME to graduate medical education based on summative entrustment decisions.

**Conclusions:**

There is emerging evidence concerning how EPAs can be effectively introduced to clinical training in UME. Specialty-specific, nested EPAs with context-adapted, entrustment-supervision scales might be helpful in better leveraging their formative assessment potential.

**Supplementary Information:**

The online version contains supplementary material available at 10.1186/s12909-021-02608-9.

## Background

Entrustable professional activities (EPAs) have been internationally implemented in graduate medical education (GME) as units of clinical activities that can be entrusted to medical trainees in the clinical workplace [1]. Given that the clinical learning trajectory begins well before entering residency, medical educators have begun to explore how to apply EPAs into undergraduate medical education (UME) as well [2]. Ideally, the intensity of supervision and, inversely, the degree of independence, should change during and with each clinical rotation of medical students. EPAs, as competency-based learning goals, take a central place within competency-based curricula in medical education. However, the rapidly growing literature on EPAs in UME has not been synthesized from the perspective of clinical educators in UME.

Globally, clinical rotations represent a foundational element in UME [3]. However, the degree of students’ active participation in both the workplace and curricular design differ considerably across medical education systems [3]. Recently, longitudinally integrated clerkships have been recommended to provide educational continuity [4]. Currently, most medical schools still work with clinical rotations that are limited to a few weeks within given specialties [5]. From an assessment perspective, using summative assessments that are limited to four- to six-week clerkships is not ideal. However, short clinical rotations do provide important formative learning and assessment opportunities for medical students [6]. It is in this context that EPAs could be used to scaffold longitudinal and formative assessment systems, even if clerkship curricula are not longitudinally integrated [7]. Assessment data points collected during these clinical rotations could also inform summative entrustment decisions within overarching assessment programs [8].

Clinical educators represent a key stakeholder group when it comes to working with EPAs in the clinical workplace. In the review by Shorey et al. [9], the authors suggested that educators engaging with competency-based medical education should consider development, implementation, and the assessment of EPAs as discrete steps, and that stakeholder specific recommendations are needed. The review of Meyer et al. [2] addressed the question of assessing EPAs in UME and revealed the inconsistent use of terms and concepts related to EPAs. Furthermore, in general, quality criteria for good assessments have not been sufficiently considered for EPAs in UME.

In light of these previous reviews, the rapid growth of literature concerning EPAs, and emerging research on the pre-clerkship use of EPAs as learning goals [10, 11] we searched the literature for articles that provided evidence on how to work with EPAs in workplace-based learning contexts in UME. Specifically, we tried to address the evidence concerning how to develop, implement, and assess EPAs in different specialties and educational systems from a clinical educator’s perspective in UME and to contrast these findings with current best practice recommendations in GME. We also aimed to highlight important research gaps that should be addressed to advance the field of competency-based education with EPAs in UME clinical training.

## Methods

We conducted a scoping review and followed the PRISMA-ScR Checklist to report our findings [12]. This approach was taken as the literature on EPAs in clerkships is still limited and emerging. Since the nomenclature for UME clinical rotations differs across educational systems, we included articles on clerkships, subinterships, and acting internships that are similar to the ‘practical year’ in Germany or the ‘elective year’ in Switzerland. Bootcamps were excluded as they typically do not include participation in daily clinical practice.

### Data sources and search strategy

Our guiding review question was: “How have EPAs been used in clinical rotations in UME with regard to development, implementation, and assessment?” We reassessed the body of literature after conducting a search of the databases: PubMed, Cochrane Library, ERIC, Embase, PsycINFO, all Ovid journals, Scopus, Web of Science, and MedEdPORTAL. The search started December 1st 2018 and went until January 15th 2019, with a search update on August 6th 2019 using the search terms [“entrustable professional activity” AND “entrustable professional activities”] for each database for publications from 2005 (the introduction of EPAs) until 2019.

### Screening and selection of articles

**Fig. 1:**
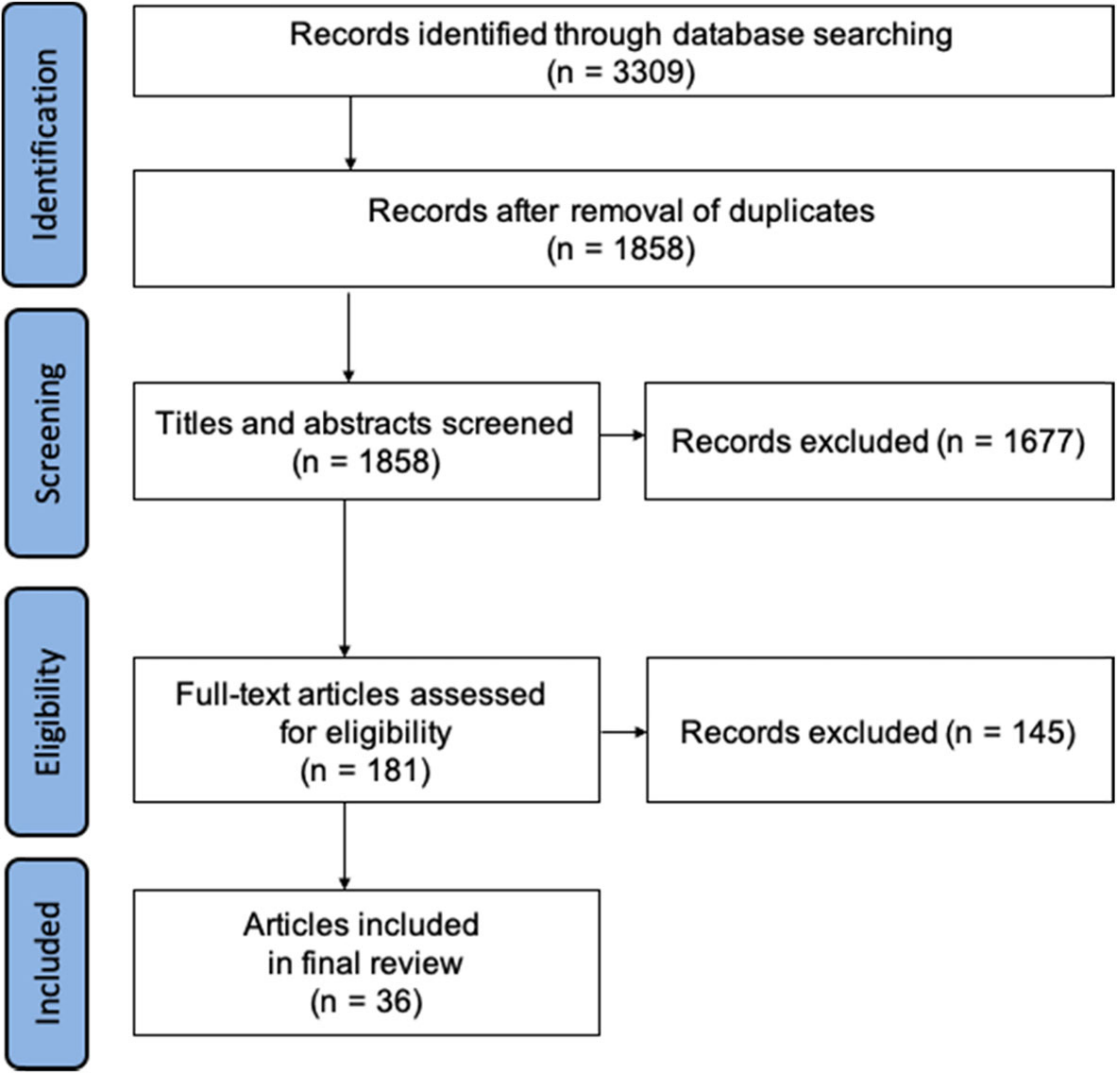
Flow-chart of the scoping review process

Inclusion and exclusion criteria are listed in Supplemental Table 1. To capture relevant EPA-based curricula in clinical workplaces in UME, we included articles related to clerkships, electives, subinternships, acting internships, and practical or transitional years. We imported all citations into EndNote X9 (Clarivate Analytics, Philadelphia, PA, USA). After the removal of duplicates, all items were screened for eligibility. First, titles and abstracts were considered, and duplicates removed (Fig. 1). We used the online software ‘Rayyan’ to select articles for full text reviews and resolve initial selection conflicts [13]. Two raters (SP and EL) independently screened all titles and abstracts in the review software. Conflicts were identified and resolved through discussion between raters. The final selection of articles was subjected to full text reviews. These were then used for data extraction and synthesis using a published data extraction form [1] to ensure comparability of our review. The final selection was cross-checked with the predefined inclusion and exclusion criteria.

### Data extraction and synthesis of results

National EPA-frameworks for UME were charted in a table and EPA content was compared (Table 2). We further applied codes for the main categories of EPA development, implementation, and assessment as described in the literature [1] (Supplemental Table 2). Codes were added iteratively. We focused on extracting data that were considered directly relevant for clinical rotation curriculum design based on the authors’ consensus (Supplemental Table 3). Since we conducted a scoping review (and due to the heterogeneous nature of the studies) we did not assess the quality of the studies.

## Results

### Search results

A total of 3309 articles were identified through database searches, and 1858 titles and abstracts were screened after the removal of duplicates. The screening resulted in 1677 articles being removed. There were 181 articles included in the full text assessment. A final selection of 36 articles was used in the data extraction. We found 20 newly published articles that had not been included in previous reviews on EPAs in the context of UME. The flow of the database search is shown in Fig. 1.

### Summary of study characteristics

The included studies were summarized according to country of origin, type of article, specialty focus, and main focus in terms of EPA development, implementation, and assessment (Table 1). All of the articles originated from American and European countries, and the studies were predominantly carried out at single academic institutions. One study was conducted as a two-center study in the Netherlands and Hungary. We found an overall increasing trend of publication quantity per year. In terms of article type, the majority were categorized as expert consensus articles followed by educational case reports and cohort studies. Most articles did not focus on a specific specialty; however, those that did were mostly in the context of internal medicine, general surgery, and emergency medicine. The studies typically focused on one or two of the following aspects: development, implementation, or the assessment of EPAs in UME clinical rotations.

**Table 1:** Summary of article characteristics (*n* = 36) included in the scoping review on entrustable professional activities in clinical rotations in undergraduate medical education

Countries of origin		USA (*n* = 22, 61%), The Netherlands (*n* = 4, 11%, one multicenter study), Canada (*n* = 4, 11%), Germany (*n* = 3, 8%), Hungary (*n* = 1, 3%, multicenter study), Australia (*n* = 1, 3%), Mexico (*n* = 1, 3%), Switzerland (*n* = 1, 3%)	
**Type of article**			
Expert consensus	*n* = 12 (33%)	Qualitative study:	*n* = 4 (11%)
Educational case report:	*n* = 6 (17%)	Mixed methods:	*n* = 4 (11%)
Cohort study:	*n* = 6 (17%)	Quasi randomized:	*n* = 1 (3%)
Survey study:	*n* = 2 (6%)	Evaluation study:	*n* = 1 (3%)
**(Sub-)Specialty focus**^**a**^			
No specialty focus		*n* = 16 (44%)	
Internal medicine		*n* = 9 (25%)	
General surgery		*n* = 7 (19%)	
Emergency medicine		*n* = 4 (11%)	
Anesthesiology		*n* = 3 (8%)	
Psychiatry		*n* = 3 (8%)	
Cardiology		*n* = 2 (6%)	
Intensive care		*n* = 2 (6%)	
Respiratory medicine		*n* = 2 (6%)	
Gynecology and Obstetrics		*n* = 2 (6%)	
Family medicine		*n* = 2 (6%)	
Neurology		*n* = 2 (6%)	
Pediatrics		*n* = 1 (3%)	
Physical medicine and rehabilitation		*n* = 1 (3%)	
**Main focus**^**b**^			
Development		*n* = 17 (47%)	
Implementation		*n* = 18 (50%)	
Assessment		*n* = 19 (53%)	

^a^Some articles covered more than one specialty. ^b^Main focus refers to whether EPA-development (both individual EPA development and EPA-curriculum framework development), EPA-curriculum implementation or EPA-based assessment was primarily addressed in the article, many articles covered more than one focus

### National EPA-frameworks for clinical curriculum design in UME

Three national organizations, including the Association of American Medical Colleges (AAMC) [17], the Association of Faculties of Medicine of Canada (AFMC) [15], and the Joint Commission of the Swiss Medical Schools (SMIFK/CIMS) [16], have published sets of EPAs that are intended to cover the full range of UME, including clinical rotations such as clerkships, subinternships, and electives. The numbers of core EPAs (those that are not specialty specific and cover a full UME program) differed between countries (13 published in 2014 in the AAMC-framework, 12 in 2016 in the AFMC-framework, and 9 in 2016 in the SMIFK/CIMS-framework). The wording of some EPA titles was slightly different in the three catalogues. A juxtaposition of the EPAs per framework is shown in Table 2.

**Table 2:** Entrustable professional activity (EPA) frameworks in undergraduate medical education (UME)

Framework organization	AAMC^a^ [14]	AFMC^b^ [15]	PROFILES^c^ [16]
Country	USA	Canada	Switzerland
Year of publication	2014	2016	2017
Number of UME EPAs	13	12	9
Corresponding EPAs (based on content)	EPA 1: Gather a history and perform a physical examination	EPA 1: Obtain a history and perform a physical examination adapted to the patient’s clinical situation	EPA 1: Take a medical history
	*see AAMC EPA 1*	*see AFMC EPA 1*	EPA 2: Assess the physical and mental status of the patient
	EPA 2: Prioritize a differential diagnosis following a clinical encounter	EPA 2: Formulate and justify a prioritized differential diagnosis	EPA 3: Prioritize a differential diagnosis following a clinical encounter
	EPA 3: Recommend and interpret common diagnostic and screening tests	EPA 3: Formulate an initial plan of investigation based on the diagnostic hypotheses	EPA 4: Recommend and interpret diagnostic and screening tests in common situations
	*see AAMC EPA 3*	EPA 4: Interpret and communicate results of common diagnostic and screening tests	*see PROFILES EPA 4*
	*see AAMC EPA 12*	*see AFMC EPA 11*	EPA 5: Perform general procedures
	*see AAMC EPA 10*	*see AFMC EPA 8*	EPA 6: Recognize a patient requiring urgent/ emergency care, initiate evaluation and management
	EPA 4: Enter and discuss orders and prescriptions	EPA 5: Formulate, communicate, and implement management plans	EPA 7: Develop a management plan, discuss orders and prescriptions in common situations
	EPA 5: Document a clinical encounter in the patient record	EPA 6: Present oral and written reports that document a clinical encounter	EPA 8: Document and present a patient’s clinical encounter; perform handover
	EPA 6: Provide an oral presentation of a clinical encounter	*see AFMC EPA 6*	*see PROFILES EPA 8*
	EPA 7: Form clinical questions and retrieve evidence to advance patient care	*no corresponding AFMC EPA*	*no corresponding PROFILES EPA*
	EPA 8: Give or receive a patient handover to transition care responsibility	EPA 7: Provide and receive the handover in transitions of care	*see PROFILES EPA 8*
	EPA 9: Collaborate as a member of an interprofessional team	*no corresponding AFMC EPA*	*no corresponding PROFILES EPA*
	EPA 10: Recognize a patient requiring urgent or emergent care and initiate evaluation and management	EPA 8: Recognize a patient requiring urgent or emergent care, provide initial management and seek help	*see PROFILES EPA 6*
	*no corresponding AAMC EPA*	EPA 9: Communicate in difficult situations	*no corresponding PROFILES EPA*
	*see AAMC EPA 13*	EPA 10: Participate in health quality improvement initiatives	EPA 9: Contribute to a culture of safety and improvement
	EPA 11: Obtain informed consent for tests and/or procedures	*no corresponding AFMC EPA*	*no corresponding PROFILES EPA*
	EPA 12: Perform general procedures of a physician	EPA 11: Perform general procedures of a physician	*see PROFILES EPA 5*
	*no corresponding AAMC EPA*	EPA 12: Educate patients in disease management, health promotion and preventive medicine	*no corresponding PROFILES EPA*
	EPA 13: Identify system failures and contribute to a culture of safety and improvement	*see AFMC EPA 10*	*see PROFILES EPA 9*

^a^*AAMC* Association of American Medical Colleges

^b^*AFMC* The Association of Faculties of Medicine of Canada

^c^*PROFILES* Principal Relevant Objectives and Framework for Integrative Learning and Education in Switzerland for the training of medical students

### EPA development for clinical rotations in UME

We identified 17 articles (47%) that explicitly addressed the development of single EPAs or EPA-based curricula for clinical rotations in UME (Table 1). National frameworks defined core EPAs for UME but did not specify what each specialty should cover in their clinical rotation—if they offered one. Several articles reported on ‘nested’ EPAs (smaller units of clinical activities that fed into core EPAs) or specialty-specific EPAs that could fit into both national frameworks and specialty-specific clinical learning environments [18–21].

Examples include “Evaluation of patients with respiratory insufficiency” [18] or “Gather a medical history, perform a physical exam and provide a structured summary of the results” [20]. Educators adjusted either nested or specifically developed EPAs for their local clinical rotation context [22–26]. All of these subsets had fewer EPAs in comparison to the national EPA-frameworks in UME.

The degree of referencing existing, relevant documents and validating EPA content, construct, and applicability of newly developed EPAs to specific clinical rotation contexts was varied. Several methods, such as the Delphi method [23], surveys [22, 23] and stakeholder interviews [10, 20] were described to increase the validity of identified clerkship EPAs, but they were not used consistently in the literature.

### EPA-based curriculum implementation

Eighteen of the 36 included articles reported on EPA-based curriculum implementation strategies in the context of clinical rotations (Table 1). These were reported at the micro-level within a clerkship curriculum, as well as higher-level implementations that covered several specialties or two educational phases (undergraduate and graduate). We found a wide range of didactic strategies used for teaching EPAs [27–30], including lectures, small-group discussions, readings, teaching rounds, and online-based learning. In terms of the implementation processes of EPA-based clinical rotations, we did not find evidence for existing best practices in the early phase that would allow for recommendations of specific curricular elements and didactic strategies per core EPA.

Several educational case reports were published that shared institution-based experiences concerning faculty development [24, 31], implemented teaching sessions [29], written or electronic EPA portfolios [32], clerkship curriculum stewardship [33], and working with clinical competency committees [30, 34, 35]. Implementing nested EPAs was one strategy used within clinical rotation contexts [34]. The implementation of longitudinal EPA-based curricula was reported to require more time and personnel resources to synthesize entrustment decisions for summative purposes [36]. This was also the case for time-variable learner handovers between educational phases and workplaces [30].

Longitudinally integrated clerkship structures appeared to be better suited for meaningfully collecting several entrustment data points for high-stakes entrustment decisions over time [18, 29, 30, 33, 37]. Traditional block clerkship curricula ranging from two to 12 weeks were, however, the predominant structure in the EPA-based clerkship curricula [20, 22–27, 32, 36, 38–44]. Two articles reported highly integrated, and longitudinally and specialty oriented, EPA-based curricula that covered both UME and GME, one in pediatrics [35] and one in internal medicine [45]. Only one article reported on the implementation of a curriculum with time-variable progression to residency (shorter or longer duration of undergraduate training based on summative entrustment decisions) [35].

In terms of implementing EPA-based clinical rotation curricula, medical students were reported to act as important change agents in the early implementation phase [29, 30]. Medical students have managed to interest their clinical preceptors in EPA-based curricula and have informed them, in a bottom-up fashion, about reformed curriculum structures. This has helped to generate buy-ins of teaching faculty. One qualitative study reported on the perceptions of teaching faculty in regard to implementing an EPA-based clinical rotation curriculum and found that faculty perceived it as potentially beneficial for patient safety through a better structure of clinical training curricula [44].

### EPA assessment in UME clinical rotations

Nineteen of the 36 included articles reported on the assessments of EPAs in clinical rotations in UME (Table 1). We found a wide range of methods, tools, and measures to assess EPAs or aspects of EPAs in terms of knowledge, skills, or attitudes.

#### Entrustment ratings and summative assessments

Educators used a variety of entrustment scales (binary [32] or different subdivisions of entrustment levels [22]) and varying target entrustment levels for clerkship students (e.g., co-activity versus indirect supervision). Educators typically adjusted published entrustment scales and rubrics [10, 34] to their local needs [23, 25]. Entrustment scales were reduced to binary entrustment decisions [32, 40] or expanded to scales subdivided into 3 to 9 levels of entrustment stages [22, 25, 46] to assess entrustability. Some studies used checklists or rating scales to assess levels of competence, but they did not report any assessment of levels of entrustability [27, 28, 38, 41]. Student self-ratings on performance scales in clinical rotations were compared to expert ratings and did not correlate well [46, 47]. Students tended to overestimate their achieved performance level in comparison to the rating given by experts. One study that compared expert ratings for one EPA did not show significant differences between entrustment ratings of experts [48].

Comparisons of self-entrustment ratings before and after clerkships were also used to identify EPAs that are frequently observed [32, 43, 46]. One article reported on a feasible assessment program that allowed for making high-stakes entrustment decisions and time-variable progress to residency [30]. Another study reported on validating an applied entrustment scale with an educational utility framework based on measures of feasibility, reliability, validity, and educational value [25].

#### Feedback on EPAs and formative assessments

An EPA-based assessment strategy within a surgical clerkship showed positive effects in the observation frequency of clinical activities and constructive feedback. The use of a binary entrustment scale had no educational effect [32]. In a study across several acute care specialties, feedback on EPAs was perceived as helpful in aligning educational activities. However, students perceived entrustment ratings as less relevant for acute-care settings, since those activities were usually carried out by residents [29]. One study provided evidence concerning the elements of high-quality feedback in the context of EPA-based clinical rotation curricula [39]. These included focused instructions for improvements based on both students’ self-reflection and expert opinion. We found no empirical studies that compared educational outcomes based on entrustment ratings or formative feedback on EPAs as compared to other assessment methods.

## Discussion

### Summary of the main results

The present scoping review summarizes the emerging evidence concerning EPAs in UME clinical rotations. In total, 36 articles, including three national core EPA frameworks, were identified and used for full data extraction. We saw an upward and international trend in the quantity of published articles. Methods for developing EPAs, implementing EPA-based clinical curricula in UME, and assessing EPAs varied and no clear standard has yet emerged for UME clinical rotations. When developing EPAs, educators should critically consider existing national frameworks, previously published, specialty-specific EPAs, or using some form of a validation process (e.g., survey, Delphi study, stakeholder interviews) for novel EPAs. The EQual rubric has been identified as a helpful tool to ensure the quality of EPA definitions in UME [49, 50].

In terms of EPA-based curriculum implementation, we did not find a gold standard. EPAs used in clinical UME differed significantly in terms of acuity, complexity, and variability both within national frameworks and between specialties (e.g., taking a psychiatric history versus managing a life-threatening emergency). As developing high-quality EPAs is only one element in the design of a clinical curriculum (mainly the goals and objectives in Kern’s six-step approach [51]), a better understanding of the effectiveness of educational and implementation strategies per core EPA is necessary.

Similarly, assessment tools and strategies for (core) EPAs in clinical UME have been used heterogeneously. We found a range of workplace-based assessments and novel entrustment-supervision scales for assessing EPAs in UME clinical training, but these might need to undergo more robust validation in light of current assessment validity frameworks [52, 53]. Overall, the nature and use of entrustment-supervision scales in undergraduate clinical training merits further exploration before recommendations can be made [54].

### National EPA-frameworks for clinical curriculum design in UME

Three national EPA-frameworks for UME have been published [14–16] and used for clerkship curriculum designs in various medical specialties (Table 1).

Despite some differences in wording and the number of core EPAs, these national frameworks help to compare national educational contexts and could provide a basis for future educational collaborations and international research projects. More research is needed before making recommendations on how to design EPA-based clinical curricula in a way that fits into national frameworks, as well as into specialty-specific contexts.

### EPA development for clinical rotations in UME

One challenge when designing clinical curricula in UME is to balance specialty-specific EPAs for undergraduate students planning to choose that specialty and core EPAs that are relevant for all medical students regardless of their chosen specialty. Different approaches to developing EPA-based curricula have been described [55, 56] for GME and could be similarly used for undergraduate clinical rotations. Data from clerkship studies [32, 47] indicate that competency progression changes differently for core EPAs. This is not surprising given the obvious differences between a clinical rotation in surgery as compared to psychiatry. However, these differences should be used to focus and align limited educational resources according to the specific clinical learning contexts instead of trying to accommodate all core EPAs in all clinical rotations. Therefore, we suggest that educators consider three stakeholder perspectives when developing EPA-based curricula for their clinical rotation: the overarching UME curriculum based on core EPAs that are not specialty specific, the specialty-specific clinical context at the level of UME, and the specialty-specific clinical context at the level of GME (residents and attendings as supervisors of medical students). Finally, new tools to evaluate the construct quality of EPAs such as the EQual score [57] should be used in UME.

### EPA-based curriculum implementation

From an implementation perspective, we found that faculty development plays an important role [18, 29–31]. Educators must be prepared to use teaching and coaching strategies that help trainees move up the entrustment scale [58]. One novel approach would be to work with medical students as part of faculty development [29]. Students have functioned as additional change agents through ad hoc explanations of EPA concepts to clinical preceptors. To implement EPA-based curricula across institutions, we see a need for a more consequential use of published EPA templates [59] that would allow for the transfer of successful implementation strategies. Furthermore, it might be challenging to effectively implement EPA-based clinical curricula together with teaching additional core skills such as time-management skills and personal well-being in clinical workplaces within 4 weeks [26]. Therefore, an integrated longitudinal clerkship curriculum design might offer more opportunities to accommodate these personal development objectives [36].

### EPA assessment in clinical rotations in UME

There was an overall heterogeneity with regard to reported educational outcome measures for EPA-based clinical curricula. Assessment methods included written knowledge-based tests [41, 48], a combination of these tests with workplace-based assessments [42], and summative and time-variable entrustment and progression decisions [30]. The articles we found addressed the assessment of EPAs primarily within a clinical rotation. Educators might consider an assessment framework that serves both the specific clinical rotation and an overarching programmatic assessment framework [8] to allow for summative entrustment decisions.

Based on our review results, the potential of (self-) assessing learning goals in the form of EPAs with entrustment-supervision levels is not clear yet. Binary entrustment scales might not yield meaningful assessment data in the context of clinical rotations [32]. The inaccuracy of the self-assessment of competence is well described [60] and has been confirmed in the context of EPAs [46, 47]. However, looking at entrustment estimation from a self-efficacy theory perspective [61] might help educators to address identified needs for more supervision per core EPA to coach students along their clinical learning trajectory.

### Limitations

This scoping review has some limitations. Due to the early phase of this emerging research field and the heterogeneity of studies, we were unable to pool data and perform a meta-analysis. Furthermore, since we limited our search to the English phrase “Entrustable professional activity” we may have missed articles from countries that use different terms (e.g., ‘Actividades Profesionales Confiables (APROC)’ in Spanish or ‘Anvertraubare professionelle Tätigkeiten (APTs)’ in German). Additionally, we searched nine databases but did not search the grey literature; thus, we might have missed relevant articles. In terms of data extraction, we focused on aspects relevant to clinical educators; therefore, other stakeholder perspectives might be underrepresented.

### Implications for practice and research

EPAs can be meaningfully used to redesign clinical curricula in UME and to focus limited educational resources on competency-based learning outcomes. Strategies to develop validated EPAs in clinical UME and tools for workplace-based assessment of EPAs are emerging. Clinical educators can build on existing national frameworks and specialty-specific EPAs to develop local curricula. Furthermore, EQual rubrics can be used for quality assurance for identifying and developing suitable EPAs [57]. High-quality EPAs should then be used as a basis for systematic clinical curriculum development [51]. A better understanding of EPA-assessment within and across clinical rotations—ideally in the context of programmatic assessment systems—is needed [8].

## Supplementary Information

**Additional file 1: Supplemental Table 1**. Inclusion and exclusion criteria.

**Additional file 2: Supplemental Table 2**. Applied codes for data extraction.

**Additional file 3: Supplemental Table 3**. Data extraction EPAs in clerkships.

## Data Availability

The datasets used and analyzed for this study are available from the corresponding author on reasonable request.

## Abbreviations

AAMC: Association of American Medical Colleges
AFMC: Association of Faculties of Medicine of Canada
EPA: Entrustable Professional Activity
UME: Undergraduate Medical Education
GME: Graduate Medical Education
PROFILES: Principal Relevant Objectives and Framework for Integrated Learning Education in Switzerland
