# Supplementary material for: Working with entrustable professional activities in clinical education in undergraduate medical education: a scoping review
Source: BMC Med Educ. 2021 Mar 19;21:172. doi: 10.1186/s12909-021-02608-9 (PMC7980680; doi:10.1186/s12909-021-02608-9)
Supplement: Supplementary file 1 — Additional file 1: Supplemental Table 1. Inclusion and exclusion criteria. [file 12909_2021_2608_MOESM1_ESM.docx]

| **Supplemental Table 1.** Inclusion and exclusion criteria | |
| --- | --- |
| Inclusion criteria | Exclusion criteria |
| English, German, Spanish or French text available | Main focus on graduate medical education (GME) |
| Focus on medical students | Focus on non-clinical educational context |
| Focus on undergraduate medical education (UME) | Focus on other health professions’ education |
| Entrustable professional activity/ies | Bootcamps for specialty specific preparation |
| Focus on clerkships, core-clerkships, subinternships, acting internship, transitional years, practical year, clinical electives | No full text available (abstracts only) |
| Clinical curriculum | Focus on milestones |
|  | Focus not on EPAs |
