# Supplementary material for: Working with entrustable professional activities in clinical education in undergraduate medical education: a scoping review
Source: BMC Med Educ. 2021 Mar 19;21:172. doi: 10.1186/s12909-021-02608-9 (PMC7980680; doi:10.1186/s12909-021-02608-9)
Supplement: Supplementary file 2 — Additional file 2: Supplemental Table 2. Applied codes for data extraction. [file 12909_2021_2608_MOESM2_ESM.docx]

| **Supplemental Table 2.** Applied codes for data extraction* | |
| --- | --- |
| **Development process** | **Implementation/Assessment codes** |
| - Literature review - Initial EPAs drafted   - From literature review   - By working group   - From interviews/focus groups - EPAs revised and refined   - Delphi Method   - Survey   - Stakeholder deliberation - EPAs mapped to milestones/competencies - Curriculum Objectives developed - EPAs grouped by specialty - Ten Cate template consulted - EPAs tested for completeness in clinical setting - EPAs merged with previously developed sets of EPAs - EPAs benchmarked with other sets of EPAs | Implementation   - Teaching Sessions - Observation of faculty on the ward - Enrolment in competency-based curriculum - Peer feedback - EPA performance recorded by trainee in portfolio   Assessment (Methods)   - EPA observed and assessed in practice by senior faculty - Non-clinical performance - Portfolio review - Chart-based audit - Comparison with control group - Written Exam   Assessment (Tools/Measures)   - Assessment form - Standardized rubric - Global entrustment scale - Number of errors - Time to entrustment |
| *Adapted from: O'Dowd, E., Lydon, S., O'Connor, P., Madden, C., & Byrne, D. (2019). A systematic review of 7 years of research on entrustable professional activities in graduate medical education, 2011–2018. Medical education, 53(3), 234-249. | |
