# Supplementary material for: Working with entrustable professional activities in clinical education in undergraduate medical education: a scoping review
Source: BMC Med Educ. 2021 Mar 19;21:172. doi: 10.1186/s12909-021-02608-9 (PMC7980680; doi:10.1186/s12909-021-02608-9)
Supplement: Supplementary file 3 — Additional file 3: Supplemental Table 3. Data extraction EPAs in clerkships. [file 12909_2021_2608_MOESM3_ESM.docx]

| **Supplemental Table 3.** Data extraction EPAs in clerkships | | | |
| --- | --- | --- | --- |
| **Authors (year of publication)/**  **Country of study/**  **Type of article** | **Subspecialty of clerkship and context** | **Title of article and applied codes^ψ^** | **Specific EPAs referenced or developed for clerkships/subinternships and main aspects reported** |
| 1) AAMC* (2014) / USA/ Expert consensus | Clerkships in UME** in general | Core entrustable professional activities for entering residency: Curriculum developers’ guide  Codes:  Development:   - Initial EPAs drafted   - From literature review   - By working group | 13 core AAMC EPAs:  1) Gather a history and perform a physical examination  2) Prioritize differential diagnosis following a clinical encounter  3) Recommend and interpret common diagnostic and screening tests  4) Enter and discuss orders and prescriptions  5) Document a clinical encounter in the patient record  6) Provide an oral presentation of a clinical encounter  7) Form clinical questions and retrieve evidence to advance patient care  8) Give or receive a patient handover to transition care responsibility  9) Collaborate as a member of an interprofessional team  10) Recognize a patient requiring urgent or emergency care and initiate evaluation and management  11) Obtained informed consent for tests and/or procedures  12) Perform general physician procedures  13) Identify system failures and contribute to a culture of safety and improvement |
| 2) Hirsh et al. (2014) / USA/ Expert consensus | Clerkships in UME in general,  Longitudinal integrated clerkships (LIC) | Time to Trust: Longitudinal Integrated Clerkships and Entrustable Professional Activities  Codes:  Implementation:   - Longitudinal clerkship structure | Observations and recommendations:   - LIC models restore educational continuity, both in trainee–patient relationships and trainee–supervisor relationships. - LICs expected to foster more robust longitudinal trainee–trainee teamwork and trainee–system work-cornerstones of quality and safety - Emerging evidence that longitudinal experiences are beneficial for both UME and GME** programs - Short rotation-based clerkships may not allow clinicians to build sufficient relationships with students to acknowledge their strengths and limitations, which is essential for entrustment decisions - Longitudinal engagement is more conducive to competency-based education with EPAs - Early experience with responsibility and developmentally oriented longitudinal oversight are key to efficient education |
| 3) AFMC*** (2016)/ Canada/ Expert consensus | Clerkships in UME in general | Entrustable professional activities for the transition from Medical School to Residency  Codes:  Development:   - Initial EPAs drafted   - From literature review   - By working group - EPAs revised and refined   - Stakeholder deliberation | 12 AFMC EPAs:  1) Obtain a history and perform a physical examination, adapted to the patient's clinical situation  2) Formulate and justify a prioritized differential diagnosis  3) Formulate an initial plan of investigation based on the diagnostic hypotheses  4) Interpret and communicate results of common diagnostic and screening tests  5) Formulate, communicate and implement management plans  6) Present oral and written reports that document a clinical encounter  7) Provide and receive the handover in transitions of care  8) Recognize a patient requiring urgent or emergent care, provide initial management and seek help  9) Communicate in difficult situations  10) Participate in health quality improvement initiatives  11) Perform general procedures of a physician  12) Educate patients on disease management, health promotion and preventative medicine |
| 4) Chen et al. (2016) / USA/ Educational case report | EPAs for entry into clerkship in general | Developing Entrustable Professional Activities  for Entry Into Clerkship  Codes:  Development:   - Initial EPAs drafted   - From literature review   - By working group   - From interviews/focus groups - EPAs mapped to milestones/competencies | 5 core EPAs for entry into clerkship:   - Gather information from a medically stable patient with a common chief complaint - Integrate information gathered about a patient to construct a reasoned and prioritized differential diagnosis as well as a preliminary plan for common chief complaints - Communicate information relevant to a patient’s care with other members of the health care team - Share information about the patient’s care, including diagnosis and management plan, with a patient in no significant physical or emotional distress - Provide the health care team with resources to improve an individual patient’s care or collective patient care   Recommendations:   - Individual institutions may choose to include additional, elective EPAs, such as one related to procedures, to suit their institution-specific objectives and student needs. - Institutions interested in implementing these core preclerkship EPAs should complete parts 3, 4a, 4b, 5, and 6 of the EPA descriptions according to their local curriculum - Multiple and preferably different types of information sources (e.g., faculty evaluation, multisource feedback, standardized patient exams) should be used to gauge progress and entrustment decisions should be based on the input of more than one person or time point (e.g., three faculty members recommending entrustment) |
| 5) Jablonover et al. (2016) / USA/ Survey study | Internal medicine clerkship | ECG as an Entrustable Professional Activity: CDIM Survey Results, ECG Teaching and Assessment in the Third Year  Codes:  Implementation   - Survey of implementation practices | 1 EPA: ECG interpretation  Reported results:   - ECG instruction was re- ported to occur in the third-year internal medicine clerkship in 85% of the schools - Ten percent of the respondents stated that ECG instruction was not taught during the internal medicine clerkship - Multiple pedagogical techniques were utilized to teach ECGs, including 62% lecture, 55% small group sessions, 30% assigned readings, 22% formal teaching rounds, and 18% Web-based modules - 37% of the respondents stated that the average number of ECGs a student was formally asked to interpret during the clerkship was >10, 24% of the respondents noted that they did not know how many ECGs a student at their school formally interpreted during the clerkship. - Nearly 100% of respondents reported that by the end of the internal medicine clerkship, every student should be able to recognize the following ECG rhythms/abnor- malities:   - Sinus rhythm, sinus tachycardia, sinus brady- cardia, atrial fibrillation, atrial flutter, first-degree atrioventricular block, complete heart block, premature ventricular complexes, ventricular tachycardia (mono- morphic), bundle branch block, left axis deviation, left ventricular hypertrophy, ST-segment elevation myocar- dial infarction, acute pericarditis, and hyperkalemia - 42% of respondents stated that their third-year internal medicine clerkship did not include any assessment of ECG interpretation - Most common method of assessment of ECG interpretation ability, utilized in the 43% of clerkships where ECG assessment occurred, was a written examination (24%) |
| 6) Norbury et al. (2016) / USA/ Educational case report | Physical Medicine and Rehabilitation clerkship (required, 2 weeks duration) | Impact of a Revised Curriculum Focusing on Clinical  Neurology and Musculoskeletal Care on a Required Fourth-Year Medical Student Physical Medicine and Rehabilitation Clerkship  Codes:  Development:   - - Using AAMC EPA Framework to guide curriculum development | 13 core AAMC EPAs  1. Gather a history and perform a physical examination  2. Prioritize differential diagnosis following a clinical encounter  3. Recommend and interpret common diagnostic and screening tests  4. Enter and discuss orders and prescriptions  5. Document a clinical encounter in the patient record  6. Provide an oral presentation of a clinical encounter  7. Form clinical questions and retrieve evidence to advance patient care  8. Give or receive a patient handover to transition care responsibility  9. Collaborate as a member of an interprofessional team  10. Recognize a patient requiring urgent or emergency care and initiate evaluation and management  11. Obtained informed consent for tests and/or procedures  12. Perform general physician procedures  13. Identify system failures and contribute to a culture of safety and improvement  Results reported:   - Partly higher students’ satisfaction with EPA-based curriculum in rotation evaluations as compared to traditional curriculum. Not significant (p < 0.05) for overall rotation rating (5-point Likert scale), significant improvement for learning objectives, didactic sessions, interviewing skills, diagnostic and patient management skills (4-point Likert scale) |
| 7) Reyes et al. (2016) / USA/ Quasi-randomized study | Internal medicine clerkship (required, 4 weeks duration) | Effect of handoff skills training for students during the medicine clerkship: a quasi-randomized Study  Codes:  Implementation:   - Teaching Sessions - Observation of faculty on the ward   Assessment (Methods)   - EPA observed and assessed in practice by senior faculty - Non-clinical performance - Comparison with control group   Assessment (Tools/Measures)   - Assessment form - Standardized rubric | 1 EPA: Patient handover  Results:   - Intervention students mean score demonstrated improvement in handoff skills immediately after the workshop (2.6–3.8; p \ 0.0001) - These results persisted into fourth year acting internship when compared to baseline performance (3.9–3.5; p = 0.06) and to untrained control students (3.5 vs. 2.5; p \ 0.001, d = 1.2) - Intervention students evaluated in the clinical setting scored higher than control students when assessed doing real-time handoffs (3.8 vs. 3.3; p = 0.032, d = 0.71) |
| 8) SMIFK/CIMS^Δ^ (2016) / Switzerland/ Expert consensus | Clerkships in UME in general | Principal Relevant Objectives and Framework for Integrated Learning and Education in Switzerland  Development:   - Initial EPAs drafted   - From literature review   - By working group | 9 SMIFK/CIMS EPAs:  1) Take a medical history  2) Assess the physical and mental status of the patient  3) Prioritize a differential diagnosis following a clinical encounter  4) Recommend and interpret diagnostic and screening tests in common situations  5) Perform general procedures  6) Recognize a patient requiring urgent / emergency care, initiate evaluation and management  7) Develop a management plan, discuss orders and prescriptions in common situations  8) Document and present patient’s clinical encounter; perform handover  9) Contribute to a culture of safety and improvement |
| 9) Barrett et al. (2017) / Australia/ Qualitative study | Internal medicine, Surgery, Anesthesiology (required, 9 weeks duration per specialty) | Novice students navigating the clinical environment in an early medical clerkship  Codes:  Development:   - EPA-curriculum development | Results:   - Finding and contacting patients challenges all students and overwhelms some - The educational design of the placement is a flawed navigational device providing inadequate clarity and security - The physical and social terrain of a large tertiary hospital is replete with obstacles making it easy for some students to stumble and retreat - Any positive connection with peers, staff and patients is empowering - Perceptions of students’ when to approach a patient:   - willingness to spend time talking with students;   - ability to speak English (well);   - not to be taking a shower or cleaning their teeth;   - not to be in pain;   - to be well enough to bother;   - to be awake;   - not to be too frail;   - to be in a good frame of mind;   - not to be attached to too much equipment;   - not to be with another clinician or carer;   - not to have ‘contact precautions’;   - not to have visitors; not to be eating a meal;   - not to be on the telephone;   - not to be fasting;   - not to be delirious;   - not to be too confused to have insight into their health problems;   - not to have just been given bad news,   - not to have been recently admitted after attempting suicide.   Recommendations:   - Medical students in early clinical clerkships require explicit education about patients, about themselves and about how to interact with the complex health care environment - Students need sound structures for their learning, individual supervision of their early practice and active affective support, particularly informal support |
| 10) Duijn et al. (2017) / The Netherlands, Hungary/ Qualitative multicentre study | Clerkship experiences in general (veterinary and medical students’ perspectives) | Am I ready for it? Students’ perceptions of meaningful feedback on entrustable professional activities  Codes:  Assessment (Tools/Measures)   - Students’ perceptions of feedback on EPAs | The medical education trigger case to elicit discussion in focus groups was breaking bad news to a patient. Two guiding questions were projected on a screen or written on a white board: (1) What do you perceive as meaningful feedback to optimally prepare for performing the presented entrustable professional activity? and (2) Which information sources should or could provide this feedback?  Students experience indicated that feedback on EPAs:   - could come from a variety of sources (such as attending, resident, peers, nurses, patients, patients’ relatives, paramedics) - should come from a supervisor who is credible, trustworthy, knows the student well, - should be delivered in a safe environment - should stress both strengths and points for improvement - should be provided immediately after the observed activity - should include instructions for follow-up   Recommendations for future research:   - better and complete understanding of the process of giving, receiving, interpreting, and using feedback as a basis for real progress toward entrustment decisions needed |
| 11) Elnicki et al. (2017) / USA/ Expert consensus | Internal medicine clerkship (curricular framework proposal) | An Entrustable Professional Activity (EPA)-Based Framework to Prepare Fourth-Year Medical Students for Internal Medicine Careers  Codes:  Development:   - EPA-Curriculum-development | 13 core AAMC EPAs  1. Gather a history and perform a physical examination  2. Prioritize differential diagnosis following a clinical encounter  3. Recommend and interpret common diagnostic and screening tests  4. Enter and discuss orders and prescriptions  5. Document a clinical encounter in the patient record  6. Provide an oral presentation of a clinical encounter  7. Form clinical questions and retrieve evidence to advance patient care  8. Give or receive a patient handover to transition care responsibility  9. Collaborate as a member of an interprofessional team  10. Recognize a patient requiring urgent or emergency care and initiate evaluation and management  11. Obtained informed consent for tests and/or procedures  12. Perform general physician procedures  13. Identify system failures and contribute to a culture of safety and improvement  Recommendations to focus subinternships on  5 EPAs considered most relevant for residency preparation in internal medicine:   - EPA 4: Enter and discuss orders - EPA: 7: Form clinical questions and retrieve evidence - EPA 8: Give or receive patient handovers - EPA 9: Collaborate in healthcare teams - EPA 10: Recognize patients requiring urgent/ emergency care |
| 12) Hamui-Sutton et al. (2017) / Mexico/ Mixed-methods study | Undergraduate internship in Surgery, Gynecology and Obstetrics, Family Medicine (required, 8 weeks per specialty) | Specific entrustable professional activities for undergraduate medical internships: a method compatible with the academic curriculum  Codes:  Development   - Literature review - Initial EPAs drafted   - From literature review   - By working group   - From interviews/focus groups - EPAs revised and refined   - Delphi Method   - Survey   - Stakeholder deliberation - EPAs mapped to milestones/competencies - Curriculum Objectives developed - EPAs grouped by specialty | 8 EPAs identified for Surgery:  1. Provide care to surgical wound  2. Detect nontraumatic acute abdominal pain  3. Attend patients with diabetic foot  4. Identify vascular pathology of the lower limbs  5. Elaborate an early diagnosis of thyroid disease  6. Identify colon or anorectal disease  7. Provide care to patients with urologic pathology  8. Participate in the surgical theatre  12 EPAs identified for Gynecology and Obstetrics:  1. Give prenatal care to pregnant patient  2. Asses and assist in the care of the patient in labor  3. Provide care and counseling to women during puerperium and breastfeeding  4. Participate in the care of pregnant women with hemorrhage  5. Elaborate early diagnosis and initial care of patients with pregnancy hypertensive disorder  6. Elaborate early diagnosis and initial care of diabetic patients during pregnancy  7. Participate in the care of pregnant women with premature rupture of membranes and premature labor threat  8. Provide care to patients with cervicovaginitis  9. Provide care to women during menopause  10. Provide care to women with abnormal uterine hemorrhage  11. Detect uterine and cervical cancer  12. Detect breast cancer in women  14 EPAs identified for Family Medicine:  1. Establish control of the patient with metabolic syndrome  2. Administer vaccines  3. Provide prenatal and postnatal care  4. Carry out control of healthy child under 5 years of age  5. Make patient’s family history/ background  6. Provide full care to patients with an infectious disease  7. Provide full care to patients with an epidemiological surveillance disease  8. Provide counseling on use of contraceptive methods  9. Detect benign prostatic hyperplasia (BPH) and prostate cancer in patients at risk  10. Detect uterine and cervical cancer (CaCu)  11. Detect neoplasm of mammary gland  12. Carry out control of patients with musculo-skeletal disease  13. Detect behavior and mood disorders  14. Detect addictions  Reported experiences:   - EPA-framework provides a standardized language that helps to evaluate student’s performance and define educational strategies efficiently and accurately |
| 13) Jonker et al. (2018) / The Netherlands/ Mixed-methods study | Subinternships in Anesthesiology, Cardiology, Emergency Medicine, Intensive Care Medicine and Respiratory Medicine (required electives, dedicated transitional year in acute care disciplines, 6-12 weeks per rotation) | Connecting undergraduate and postgraduate medical education through an elective EPA-based transitional year in acute care: an early project report  Codes:  Development:   - Initial EPAs drafted   - By working group - EPAs revised and refined   - Stakeholder deliberation   Implementation:   - Teaching Sessions - Observation of faculty on the ward - Enrolment in competency-based curriculum - EPA performance recorded in portfolio - Longitudinal coach | EPA-based curriculum implementation (3 acute care EPAs):  1. Recognition and initial treatment of patients with vital instability  2. Evaluation of patients with respiratory insufficiency  3. Evaluation of patients with circulatory insufficiency  Reported experiences:   - n = 47 (enrolled students) - Early specialty choice seems to be relevant for choosing the longitudinal curriculum - Content focus on acute care perceived as strength - The attainment of EPAs at the undergraduate level of near-independence, may help to justify decisions to delegate tasks in the light of patient safety |
| 14) Klapheke et al. (2017) / USA/ Cohort study | General clerkship experiences at one institution | Third- and Fourth-Year Medical Student Self-Assessment in Entrustable Professional Activities  Codes:  Assessment (Tools/Measures)   - Assessment form - Standardized rubric (narrative descriptions of anchored ratings of 1 through 5 which described performances typically expected of first- through fourth-year medical students, Ratings 1 through 3 described progressive steps—rough milestones—in development toward competence and entrustment. Rating 4 described an entrustable student, and rating 5 described a student or resident with development of competence beyond the entrustment level) | Changes in self-assessment of performance in 13 AAMC EPAs (Core EPAs):  1. Gather a history and perform a physical or mental examination  2. Prioritize a differential diagnosis following a clinical encounter  3. Recommend and interpret common diagnostic and screening tests  4. Enter and discuss orders and prescriptions  5. Document a clinical encounter in the patient record  6. Provide an oral presentation of a clinical encounter  7. Form clinical questions and retrieve evidence to advance patient care  8. Give or receive a patient handover to transition care responsibility  9. Collaborate as a member of an inter-professional team  10. Recognize a patient requiring urgent or emergent care and initiate evaluation and management  11. Obtain informed consent for tests and/or procedures  12. Perform general procedures of a physician  13. Identify system failures and contribute to a culture of safety and improvement  Reported results (n = 30):   - At the end of the third year, 53.4 to 86.7% of students assessed themselves as entrustable (rating 4 or 5) on 5 of the 13 EPAs, with percentages self-assessing as entrustable ranging from 20.0% on EPA 4, enter and discuss orders and prescriptions, to 86.7% on EPA 9, collaborate as a member of an inter-professional team - At the end of the fourth year 66.6 to 100.0% of students rated themselves as entrustable on 11 of the EPAs (lowest ratings for EPA 4, enter and discuss orders and prescriptions (46.6%), and EPA 12, perform general procedures of a physician (53.3%) - No significant self-assessment rating increase for EPA 4, enter and discuss orders and prescriptions (p = 0.06), and EPA 11, obtain informed consent for tests and/or procedures (p = 0.42) - Greatest mean difference (0.7) in self-assessments between the third and fourth years occurred with EPA 3, recommend and interpret common diagnostic and screen- ing tests, and EPA 13, identify system failures and con- tribute to a culture of safety and improvement - Internal consistency as measured by Cronbach’s alpha showed strong values of 0.90 (third year) and 0.87 (fourth year) on self-assessments |
| 15) Klapheke et al. (2017) / USA/ Cohort study | Psychiatry clerkship (required, 6 weeks duration) | Assessing Entrustable Professional Activities During the Psychiatry Clerkship  Codes:  Assessment (Methods)   - Implementation: EPA performance - Non-clinical performance - Written Exam   Assessment (Tools/Measures)   - Standardized rubric | 8 EPAs (selected from AAMC core EPAs):  1. (EPA #1) Gather a history and perform a physical or mental examination  2. (EPA #2) Prioritize a differential diagnosis following a clinical encounter  3. (EPA #3) Recommend and interpret common diagnostic and screening tests  4. (EPA #5) Document a clinical encounter in the patient record  5. (EPA #6) Provide an oral presentation of a clinical encounter  6. (EPA #7) Form clinical questions and retrieve evidence to advance patient care  7. (EPA #9) Collaborate as a member of an inter-professional team  8. (EPA #10) Recognize a patient requiring urgent or emergent care and initiate evaluation and management  Reported results (n = 88):   - Mean pre-clerkship self-assessment ratings ranged from 2.9 to 3.5 on 5-point Likert entrustment scale, with the highest rating for EPA 9, Collaborate as a member of an inter-professional team - Mean self-assessment ratings increased significantly from pre-clerkship to post-clerkship by approximately 0.7 to 0.9 units on the rating scale, representing large effect sizes from 0.83 to 1.13, The largest mean rating increase was observed for EPA 2, Prioritize a differ- ential diagnosis following a clinical encounter - Nearly all of the post- clerkship ratings selected by students were at or above level 3 - Mean post-clerkship self-assessment ratings were significantly higher than mean post-clerkship clerkship director ratings for 7 of the eight EPAs. Mean differences associated with the largest effect size of 0.93 were observed for EPA 2 |
| 16) Pereira et al. (2017) / USA/ Expert consensus | General clerkship grading in the framework of EPAs | Criterion-Based Assessment in a Norm-Based World: How Can We Move Past Grades?  Codes:  Assessment (Methods)   - Assessment-theory - Assessment-system | Recommendation to focus on development of assessments that allow for more accurate prediction of clinical performance. Furthermore, to transition from norm-based assessment on criterion-based assessment with efficient learner handover along the learning trajectory. |
| 17) Thompson et al. (2017) / USA/ Expert consensus | Emergency Medicine clerkship (required, 4 weeks duration) | Development of an Assessment for Entrustable Professional Activity (EPA) 10: Emergent Patient Management  Codes:  Assessment   - Tool (case-based high-fidelity simulation) - Entrustment rating (global yes-/no entrustment evaluation) | AAMC EPA-10: Recognize a patient requiring urgent or emergent care and initiate evaluation and management.  Reported results:  Interrater-reliability (Krippendorf ‘s alpha) for faculty agreement on global entrustment per case: 0.53 – 1.00  87% of students were rated as having reached ad-hoc entrustment as defined by EPA-10 criteria (86.8%; 99 of 114) |
| 18) Andrews et al. (2018)/ USA/ Educational case report | General clerkship EPA-based curriculum in different settings (longitudinal integrated clerkship (LIC) and combination of block clerkships and continuity clinic) | Education in Pediatrics Across the Continuum (EPAC): First Steps Toward Realizing the Dream of Competency-Based Education  Implementation   - Students as change agents - Specialty specific curriculum lead - Clinical competency committee   Assessment (Tools/Measures)   - Assessment form - Standardized rubric - Number of errors - Time to entrustment - Target level of supervision - Published supervision scale - Validated assessment instruments | 13 Core Entrustable Professional Activities for Entering Residency (Core EPAs)  1. Gather a history and perform a physical examination  2. Prioritize a differential diagnosis following a clinical encounter  3. Recommend and interpret common diagnostic and screening tests  4. Enter and discuss orders and prescriptions  5. Document a clinical encounter in the patient record  6. Provide an oral presentation of a clinical encounter  7. Form clinical questions and retrieve evidence to advance patient care  8. Give or receive a patient handover to transition care responsibility  9. Collaborate as a member of an inter-professional team  10. Recognize a patient requiring urgent or emergent care and initiate evaluation and management  11. Obtain informed consent for tests and/or procedures  12. Perform general procedures of a physician  13. Identify system failures and contribute to a culture of safety and improvement  Experiences reported:   - 48 students enrolled - Preliminary positive curriculum feedback from students - Time-variable progression to residency possible |
| 19) Curran et al. (2018) / Canada/ Mixed-methods program evaluation study | Surgery core clerkship (required, 8 weeks duration) | Evaluation of the Characteristics of a Workplace Assessment Form to Assess Entrustable Professional Activities (EPAs) in an Undergraduate Surgery Core Clerkship  Codes:  Implementation   - Teaching Sessions - Observation of faculty on the ward - Enrolment in competency-based curriculum - EPA performance recorded by trainee in portfolio (collation of summative assessments through office of undergraduate medical education with longitudinal and regular review of performance)   Assessment:   - Tools/measures (Clinic cards to assess EPAs with a binary rating scale of either “pre-entrustable” or “entrustable”) | 9 AAMC EPAs, adopted for Core Surgery Rotation  EPA 1: Gather a history and perform a physical examination  EPA 2: Prioritize a differential diagnosis following a clinical encounter  EPA 3: Recommend and interpret common diagnostic and screening tests  EPA 6: Provide an oral presentation of a clinical encounter  EPA 7: Form clinical questions and retrieve evidence to advance patient care  EPA 10: Recognize a patient requiring urgent or emergent care, initiate evaluation and management  EPA 11: Obtain informed consent for tests and/or procedures  EPA 12: Perform general procedures of a physician  EPA 13: Identify system failures and contribute to a culture of safety and improvement  Reported results:  Statistically significant increase of entrustment based on clinic card assessments for some EPAs observed (range of percentage of average entrusted EPAs from 62% (EPA 11) to 89% (EPA 1 and EPA 6). Students and preceptors reported major challenges with regards to interpretation of EPAs’ content and of binary entrustment scale. |
| 20) Fazio et al. (2018)/ Canada/ Survey study | Internal medicine core clerkship (required, 6- to 12-weeks) | Competency-Based Medical Education in the Internal Medicine Clerkship: A Report From the Alliance for Academic Internal Medicine Undergraduate Medical Education Task Force  Development:   - Literature review - Initial EPAs drafted   - From literature review   - By working group - EPAs revised and refined   - Survey   Assessment:   - Assessment form (adjusted scale: not allowed to practice,” to “allowed under full supervision,” to “allowed with on-demand supervision.” - Standardized rubric | Development of 6 key EPAs for internal medicine core clerkship:   - Generating a differential diagnosis - Obtaining a complete and accurate history and physical exam - Obtaining focused histories and clinically relevant physical exams - Preparing an oral presentation - Interpreting the results of basic diagnostic studies - Providing well-organized clinical documentation   Perceived benefits:   - Common language across the continuum of medical education - Opportunities for cross-clerkship and cross-institutional learning and assessment collaborations   Perceived challenges:   - Time restriction of clerkships not compatible with longitudinal assessments - Limited time for direct observation and feedback in clinical workplace |
| 21) Horak et al. (2018) / USA/ Expert consensus | Neurology clerkship, general recommendations | Entrustable professional activities - A useful concept for neurology education  Development:   - EPA-Curriculum-development - EPAs mapped to specialty specific competency framework   Assessment:   - Assessment form - Standardized rubric | Examples for neurology specific EPAs in clerkships:   - Taking a neurologic history and performing the neurologic examination - Generating a differential diagnosis for common neurologic chief complaints - Interpreting common neurologic diagnostic studies   Recommendations to develop and implement EPAs in neurology across UME and GME. Mapping of relevant stakeholder groups in the context of clinical training in neurology for EPA-curriculum development. |
| 22) Jonker et al. (2018) / The Netherlands/ Qualitative study | Subinternships in Anesthesiology, Cardiology, Emergency Medicine, Intensive Care Medicine and Respiratory Medicine (required electives, dedicated transitional year in acute care disciplines, 6-12 weeks per rotation) | An elective entrustable professional activity-based thematic final medical school year: an appreciative inquiry study among students, graduates, and supervisors  Implementation   - Perception of EPA-based curriculum (students’, graduates’, supervisors’ and mentors’ perspectives) | EPA-based curriculum implementation (3 acute care EPAs):  1. Recognition and initial treatment of patients with vital instability  2. Evaluation of patients with respiratory insufficiency  3. Evaluation of patients with circulatory insufficiency  Reported results:   - Relatedness: Students appreciated evolving sense of belonging to a clinical training peer group - Content focus of EPA-based curriculum is a reason for choosing the curriculum - Students faced limited possibilities to get hands-on clinical experience in the EPAs - Thorough faculty training difficult to achieve |
| 23) Kukulski et al. (2018) / USA/ Cohort study | Emergency Medicine clerkship (required, 4 weeks duration) | Ultrasound for Volume Assessment in Patients with Shock: Effectiveness of an Educational Intervention for Fourth-year Medical Students  Implementation:  - Teaching Sessions  Assessment (Methods):   - Non-clinical performance - Written exam | 1 specialty specific EPA: Utilize inferior vena cava ultrasound measurement and echocardiography to assist in volume assessment of patients presenting with shock.  On a knowledge test, students scored an average of 45.6% (SD: 23.6, n = 83) on the pre-test and 66.4% (SD: 22.1 n = 72) on the post-test, p < 0.01 (degrees of freedom (df) = 153, t = 5.7), Cohen's d = 0.92. The satisfaction survey showed that 97.6% of students felt the session was worthwhile, 96.4% would recommend it to other students, and 83.1% felt it taught new information. |
| 24) Lupi et al. (2018)/ USA/ Expert consensus | General clerkship faculty development | Faculty Development Revisited: A Systems-Based View of Stakeholder Development to Meet the Demands of Entrustable Professional Activity Implementation  Implementation:   - Faculty development for EPA-based curricula | Requisite stakeholder skills and knowledge for EPA implementation for longitudinal clinical supervisors and clinical course directors in UME:   - Teacher improvement: WBA and DOCS/ Feedback and coaching/ Narrative comments/ Self-reflective practice/ In-depth knowledge of specific EPAs/ New skills to support teaching of specific EPAs - Leadership and management: Forward feeding, learner handovers/ Implementing systems to support quality care and clinical education/ Coordination and integration of curriculum - Research capacity building: not applicable - Academic career building: Orientation to roles and culture - Organizational change: Understanding of entrustment/ Forward feeding, learner handovers |
| 25) McClintic et al. (2018) / USA/ Cohort study | Surgery core clerkship (required, 8 weeks duration) | Curricular Innovation in the Surgery Clerkship: Can Assessment Methods Influence Development of Critical Thinking and Clinical Skills?  Development:   - Institution based adjustment of EPAs   Implementation:   - EPA-Curriculum-development - Perception of EPA-based curriculum (students’)   Assessment (Methods):   - Non-clinical performance (single OSCE) - Written exam (Pretest and Posttest Short Answer (SA) exam, knowledge-based exam scores (Shelf)) - EPAs observed and assessed in practice by senior faculty (clinical evaluation score) | Adjustment of 13 core AAMC EPAs to local curriculum, with following overarching learning objectives mapped to EPAs (corresponding EPAs in parenthesis):   - 1) Gather a history and perform a physical examination, develop a prioritized differential diagnosis with a working diagnosis, recommend and interpret appropriate diagnostic tests for a real or simulated patient with the following conditions (corresponding to EPAs 1, 2, 3) - 2) Evaluate a wound, describe the wound accurately, and recommend appropriate treatment or referral (corresponding to EPAs 1, 2, 12) - 3) Given a real or simulated patient hospitalized for a surgical condition (corresponding to EPAs 4, 5, 6):   - Provide documentation of rounding encounter in a SOAP note format   - Provide a concise, accurate oral presentation of daily rounds encounter   - Write/enter appropriate orders for the patient’s condition, either on paper or in the electronic medical record - 4) Formulate a clinical question around the presentation, workup or treatment of a surgical patient, retrieve and synthesize evidence to present a clear answer to the question (corresponding to EPA 7) - 5) Contribute as a professional member of the surgical health care teams as expected for a third-year medical student (corresponding to EPAs 8, 9, 11, 13) - 6) Recognize a patient requiring urgent or emergent care, initiate evaluation and treatment, and seek appropriate assistance (corresponding to EPA 10) - 7) Perform the procedures of a general physician (corresponding to EPA 12)   Evaluation results:   - On average the intervention group performed about 10% worse on the shelf exam - The intervention group (EPA-based curriculum) scored significantly lower on their clinical evaluations by faculty and residents - In the intervention group, short answer scores improved 40% from beginning to end of the term (as compared to 14% in control group with higher pre-test scores) - Students in the control group reported wasted down time and unfair grading, Students in the intervention group reported feeling more prepared for clinic and that portfolios facilitated critical thinking but were too time consuming. |
| 26) Menezes et al. (2018) / Canada/ Expert consensus | Psychiatry clerkship EPA-based curriculum design in general | Development:   - Literature review - EPAs mapped to milestones/competencies - Framework   Implementation (recommendations):   - Enrolment in competency-based curriculum - EPA performance recorded by trainee in portfolio   Assessment (methods recommendation)   - EPA observed and assessed in practice by senior faculty - Non-clinical performance - Portfolio review - Written Exam | Curriculum recommendations based on AFMC 12 UME EPAs:  1. Obtain a history and perform a physical examination, adapted to the patient's clinical situation  2. Formulate and justify a prioritized differential diagnosis  3. Formulate an initial plan of investigation based on the diagnostic hypotheses  4. Interpret and communicate results of common diagnostic and screening tests  5. Formulate, communicate and implement management plans  6. Present oral and written reports that document a clinical encounter  7. Provide and receive the handover in transitions of care  8. Recognize a patient requiring urgent or emergent care, provide initial management and seek help  9. Communicate in difficult situations  10. Participate in health quality improvement initiatives  11. Perform general procedures of a physician  12. Educate patients on disease management, health promotion and preventative medicine |
| 27) Ming et al. (2018) /  USA/ Cohort study | Subinternship in general | Discharge summary training curriculum: a novel approach to training medical students how to write effective discharge summaries  Codes:  Development   - Initial EPAs drafted (by working group) - From literature review   Implementation   - Teaching Sessions   Assessment (Methods)   - EPA observed and assessed in practice by senior faculty   Assessment (Tools/Measures)  - Assessment form  - Standardized rubric | 1 adapted EPA: Writing a discharge summary (corresponding to AAMC EPA 8)  Reported results:  self-/ vs expert-rating, knowledge-test,  Discharge Summary exercise; feedback  External raters (physicians) found past medical history, functional status, code status or reason for medication changes as missing in more than 20.0% of discharge summaries.  Inpatient attendings rated 95.8% (n = 23) of evaluated students as entrustable versus external raters (physicians) rated 58.3% (n = 14) of students as entrustable (p = 0.0027) on a binary entrustment scale. |
| 28) Murray et al. (2018) /  USA/ Educational case report | General clerkship EPA-based curriculum in different settings (longitudinal integrated clerkship (LIC) and combination of block clerkships and continuity clinic) | Crossing the Gap: Using Competency-Based Assessment to Determine Whether Learners Are Ready for the Undergraduate-to-Graduate Transition  Implementation   - Teaching Sessions - Observation of faculty on the ward - Enrolment in competency-based curriculum - Peer feedback - EPA performance recorded by trainee in portfolio - Specialty specific curriculum lead (pediatrics)   Assessment (Methods)   - EPA observed and assessed in practice by senior faculty - Portfolio review (through clinical competency committees (CCCs) convening every three to six months) - EPA assessments electronically recorded   Assessment (Tools/Measures)   - Assessment form (including narrative comments from standard evaluations, preceptors’ verbal observations, transcribed comments from multipreceptor sessions evaluating students on a reporter-interpreter- manager-educator (RIME) framework, student EPA self-assessments, and EPAC (Education in Pediatrics Across the Continuum Study Group)-specific EPA assessments) - Standardized rubric (adjusted) - Global entrustment scale - Number of errors - Time to entrustment (EPA threshold for UME to GME transition: Allowed to practice EPA only under reactive/on-demand supervision, with supervisor immediately available) | 13 Core Entrustable Professional Activities for Entering Residency (Core EPAs) with site-specific adaptions:  1. Gather a history and perform a physical examination. (H&P)  2. Prioritize a differential diagnosis following a clinical encounter. (DDx)  3. Recommend and interpret common diagnostic and screening tests. (Tests)  4. Enter and discuss orders and prescriptions. (Orders)  5. Document a clinical encounter in the patient record. (Document)  6. Provide an oral presentation of a clinical encounter. (Present)  7. Form clinical questions and retrieve evidence to advance patient care. (EBM)  8. Give or receive a patient handover to transition care responsibility. (Handover)  9. Collaborate as a member of an interprofessional team. (IPC)  10. Recognize a patient requiring urgent or emergent care and initiate evaluation and management. (Emergent)  11. Obtain informed consent for tests and/or procedures. (Consent)  12. Perform general procedures of a physician. (Procedures)  13. Identify system failures and contribute to a culture of safety and improvement. (Safety)  Main results reported:   - Sample size (students): n = 13 - Early evidence across the first cohort suggests a more rapid advancement in supervision levels for learners in the LICs - Students were successfully given the primary responsibility for obtaining the EPA assessments from their clinical supervisors - EPA assessment frequency ranging from an average of 1.4 to 4.2 assessments per week - Assessments for EPAs 3, 4, and 10–13 were collected less often and needed additional facilitation - EPA 12 progression to GME scale minimum needed to be modified to “transition-ready” because of lack of procedural practice opportunities for students - Time-variable transition from UME to GME is possible   Perceived barriers to EPA-based curriculum implementation:   - Lack of targeted faculty development on the assessment framework - Administrative time to summarize narrative assessments in preparation for the CCC meetings - Frequent faculty turnover and high student-to-faculty ratios |
| 29) ten Cate et al. (2018) / The Netherlands/ Educational case report | General clerkship EPA-based curriculum, longitudinal integrated clerkship (LIC) structure | The EPA-based Utrecht undergraduate clinical curriculum: Development and implementation  Development   - Literature review - Initial EPAs drafted   - From literature review   - By working group - EPAs revised and refined   - Stakeholder deliberation   Implementation   - Teaching Sessions - Observation of faculty on the ward - Enrolment in competency-based curriculum - EPA performance recorded by trainee in portfolio (electronic programmatic assessment platform)   Assessment (Methods)   - EPA observed and assessed in practice by senior faculty - Non-clinical performance - Portfolio review - Written Exam   Assessment (Tools/Measures)   - Standardized rubric - Global entrustment scale (adjusted UME scale   - 1. Permission to be present not to enact the EPA.   - 2. Direct supervision. Supervisor is present in the room. a. EPA may be conducted as a co-activity with supervisor b. EPA may be conducted alone   - 3. Indirect supervision. Supervisor is not in the room but in the immediate vicinity and quickly available for reactive/on-demand supervision. a. Supervisor will repeat the activity b. Supervisor will double check key findings and decisions c. Supervisor will double check findings within 24 h or earlier on the student’s request) | 5 core EPAs for UME   - 1) The clinical consultation - 2) General medical procedures - 3) Informing, advising and guiding patients and families - 4) Communicating and collaborating with colleagues - 5) Extraordinary patient care   Nested EPAs for UME clerkships   - 1.1 General history and physical examination including vital signs - 1.2 Gynecology clinical consultation - 1.3 Clinical consultation of pregnant woman - 1.3 Clinical consultation of neonate and infant - 1.4 Pediatric clinical consultation - 1.6 Clinical genetics consultation - 1.7 Neurological clinical consultation - 1.8 Psychiatric clinical consultation - 1.9 Geriatric clinical consultation - 1.10 Consultation for brief-episode issues in primary care - 1.11 Follow-up consultation in protocolized chronic primary care - 1.12 Public health consultation - 1.13 Clinical consultation into the out-patient setting - 1.14 Consultation of the hospital-admitted critically ill patient - 1.15 Peri-operative patient consultation - 1.16 Consultation in the acute-care setting - 2.1 Venepuncture - 2.2 Peripheral intravenous line placement and connection - 2.3 Participation in the OR - 2.4 Speculum/vaginal examination - 2.5 Insertion of urinary catheter - 2.6 Intramuscular, intra- and subcutaneous-injection - 2.7 Rectal examination - 2.8 Wound care - 3.1 Discussing diagnostic options and obtaining informed consent - 3.2 Discussing test results, prognosis, and a management plan - 3.3 Discharge conversation - 4.1 Patient handover documentation and presentation - 4.2 Acting in interprofessional teams - 5.1 Establishing death - 5.2 Basic life support   Main experiences and recommendations:   - EPAs of undergraduate education should not be small and many, but broad and few - Nest smaller clerkship EPAs within EPA-based UME curriculum - Regular grades in clinical clerkships were abandoned, but the possibility to identify extraordinary excellence and students at risk was kept - The novelty of a different organization of clerkships, a new system of assessment and the use of an electronic portfolio requires extensive faculty development - Students can be ambassadors of an EPA-based curriculum (feeling of ownership of their e-portfolio and their whole learning process, including how to pre- pare to receive entrustment for EPAs) |
| 30) Colbert-Getz et al. (2019)/ USA/ Evaluation study | Clerkship students‘ evaluations in Family Medicine, General Surgery, Internal Medicine, Neurology, Obstetrics and Gynecology, Pediatrics, and Psychiatry (required) | To What Degree Are the 13 Entrustable Professional Activities Already Incorporated Into Physicians’ Performance Schemas for Medical Students?  Assessment   - Mapping written evaluations to EPA-framework | Mapping of clinical evaluation comments to 13 AAMC EPAs (Core EPAs) before any formal introduction of EPAs in clerkships:  1. Gather a history and perform a physical examination  2. Prioritize a differential diagnosis following a clinical encounter  3. Recommend and interpret common diagnostic and screening tests  4. Enter and discuss orders and prescriptions  5. Document a clinical encounter in the patient record  6. Provide an oral presentation of a clinical encounter  7. Form clinical questions and retrieve evidence to advance patient care  8. Give or receive a patient handover to transition care responsibility  9. Collaborate as a member of an inter-professional team  10. Recognize a patient requiring urgent or emergent care and initiate evaluation and management  11. Obtain informed consent for tests and/or procedures  12. Perform general procedures of a physician  13. Identify system failures and contribute to a culture of safety and improvement  Main reported results and recommendations:   - 1,288 clinical performance evaluation forms screened - Although both strength and improvement comments were required for each form, 19 forms (1.5%) had no strength comments, 237 (18%) forms had no improvement comments - EPAs 1, 5, 6, 12, and 13 appeared in comments on 68% (703) of the forms - EPA 2–4 and 7–11 appeared in comments on 60% (616) of the forms - Most frequent EPAs addressed as either a strength or area for improvement were EPA 9 (“Collaborate as a member of an interprofessional team”; 30%, or 314), EPA 6 (“Provide an oral presentation of a clinical encounter”; 27%, or 281), EPA 2 (“Prioritize a differential diagnosis following a clinical encounter”; 25%, or 259), EPA 1 (“Gather a history and perform a physical examination”; 22%, or 225), and EPA 5 (“Document a clinical encounter in the patient record”; 15%, or 155) - EPA 4 (“Enter and discuss orders and prescriptions”) and EPA 11 (“Obtain informed consent for tests and/or procedures”) were not mentioned in any of the comments - Proportions of comment types for EPA specific strength (8%) compared to improvement (30%) was significantly different (Z= -14.78, p< .001), with a medium effect size (r= .33) - Even with no training on the 13 EPAs, physician assessors referred specifically to the activities repre- sented by the EPAs in their comments on slightly more than half of the clinical performance evaluation forms - Most relevant EPAs for core clerkships (potentially with adjustment to specialties) seem to be:   - Interviewing a patient (1a)   - Performing a physical exam (1b)   - Prioritizing and selecting a diagnosis (2a)   - Creating an assessment and plan (2b) |
| 31) Czeskleba et al. (2019)/ Germany/ Qualitative study | Subinternship in Internal Medicine and Surgery (Practical Year, i.e. the final year of medical school in the German medical education system) | Patient safety during final-year clerkships: A qualitative study of possible error sources and of the potential of Entrustable Professional Activities  Implementation   - Patient-safety in EPA-based curricula | Clinical educators’ perceptions of EPA-based clerkship curricula with regards to patient safety.  Main reported results:   - Most intensively discussed problems relate to a lack of structure of the clerkship - Inaccurate self-assessment of students's own abilities as a potential cause for avoidable adverse events - Problems can arise if assigned tasks are inappropriate or cannot be fully verified - Careless comments by students may have a significant influence, such as the willingness to undergo further care on the part of the patient - Discussion on the influence of EPAs on patient safety shows a consistently positive response among supervising physicians - Supervising physicians expect that the use of EPAs would in particular lead to a more structured clerkship - EPAs perceived as helping to provide an overview of each student’s performance level over the course of the clerkships - EPAs perceived as making the entire final-year clerkship more transparent in terms of expected learning outcomes - A concrete description of the required (and thus permitted) tasks of clerkship students together with the necessary level of supervision would give supervising physicians the confidence to know which activities they can delegate to final- year clerkship students |
| 32) Evans et al. (2019)/ USA/ Expert consensus | General longitudinal clerkships based on EPAs | Continuity in Undergraduate Medical Education: Mission Not Accomplished  Implementation   - Longitudinal clerkship structure | Evidence-oriented recommendations:   - EPA-based clerkship curricula need to be organized longitudinally in order to teach competence in chronic care - Longitudinal clerkships offer the type of relationship building over time that supports grounded trust and therefore entrustment decisions - Direct observation of history taking and physical exam skills (EPA#1); recommending screening tests at the right time, for the right patient (EPA#3); and contributing to the culture of safety and improvement (EPA#13) require making entrustment decisions about student behavior over time |
| 33) Holzhausen et al. (2019) / Germany/ Mixed-methods study (Delphi study) | General EPA development for UME clerkships at one institution | Development of Entrustable Professional Activities for  entry into residency at the Charité Berlin  Development:   - Initial EPAs drafted   - From literature review   - By working group   - From interviews/focus groups - EPAs revised and refined   - Delphi Method | 12 EPAs identified:   - 1. Along the clinical encounter   - 1.1 Gather a medical history, perform a physical exam and provide a structured summary of the results   - 1.2 Compile a diagnostic work plan and initiate implementation   - 1.3 Interpret test results and initiate further steps   - 1.4 Compile a treatment plan and initiate implementation - 2. General medical procedures   - 2.1 Perform general procedures of a physician - 3. Communication with patients   - 3.1 Seek consent for medical examinations and procedures   - 3.2 Inform and advise a patient - 4. Communication and collaboration with colleagues   - 4.1 Present a patient history   - 4.2 Give or receive a patient handover   - 4.3 Write and distribute a patient report - 5. Patient care in special situations   - 5.1 Recognize an emergency situation and act upon it   - 5.2 Undertake an evidence-based patient case presentation and initiate patient-specific implementation   Reported metrics:   - Response rates were 80, 78 and 76 % in the three Delphi rounds with 36, 35 and 34 faculty members participating in each respectively - Threshold for consent was reaching a content validity index (CVI) of ≥80% |
| 34) Keeley et al. (2019) / USA/ Educational case report | General clerkship-to-postclerkship transition | Moving Toward Summative Competency Assessment to Individualize the Postclerkship Phase  Implementation:   - Observation of faculty on the ward - Enrolment in competency-based curriculum - EPA performance recorded in portfolio - Faculty development for EPA-based curricula - Longitudinal coach   Assessment (Methods)   - EPA observed and assessed in practice by senior faculty (and resident physicians) - Narrative comments - Portfolio review (master assessors, E-portfolio) - Summative assessments (at the transitions from preclerkship to clerkship and from clerkship to postclerkship)   Assessment (Tools/Measures)   - Standardized rubric - Global entrustment scale | EPAs 1, 2, 3, 5, and 6 (from AAMC EPAs core EPAs)  1 Gather a history and perform a physical examination  2. Prioritize a differential diagnosis following a clinical encounter  3. Recommend and interpret common diagnostic and screening tests. (Tests)  5. Document a clinical encounter in the patient record  6. Provide an oral presentation of a clinical encounter  used for clerkship phase to individualize fourth year curriculum.  Reported experiences:   - Implementation of Core EPA assessments has driven a revision in the clerkship-specific required procedures and skills to avoid redundancy with Core EPA tasks and a revision of the clinical evaluation forms completed by residents and faculty. - Students below expected entrustability need to participate in remediation activities, which involve more structured scheduling with additional assessment in the early postclerkship phase before enrolling in an acting internship rotation |
| 35) Peters et al. (2019) / Germany/ Cohort study | Subinternship in Internal Medicine, Surgery and an elective (Practical Year, i.e. the final year of medical school in the German medical education system) | Introducing an assessment tool based on a full set of end-of-training EPAs to capture the workplace performance of final-year medical students  Assessment (Methods)   - EPA observed and assessed in practice by senior faculty (and resident physicians) - Narrative comments   Assessment (Tools/Measures)   - Tool development (literature review, working group) - Standardized rubric - Global entrustment scale (6-point scales for self-assessment and supervisor assessment) | Institution-based set of 12 UME end-of-training EPAs (including 72 nested EPAs listed in supplemental material of original publication)   - 1.1 Gather a medical history, perform a physical exam and provide a structured summary of the results - 1.2 Compile a diagnostic work plan and initiate implementation - 1.3 Interpret test results and initiate further steps - 1.4 Compile a treatment plan and initiate implementation - 2.1 Perform general procedures of a physician - 3.1 Seek consent for medical examinations and procedures - 3.2 Inform and advise a patient - 4.1 Present a patient history - 4.2 Give or receive a patient handover - 4.3 Write and distribute a patient report - 5.1 Recognize an emergency situation and act upon it - 5.2 Undertake an evidence-based patient case presentation and initiate patient-specific implementation   Main results:   - n = 60 (students) - Subscale reliability (McDonald’s omega for students self-assessment and supervisors assessment): good for 11 of the 12 EPAs. It is con- sidered insufficient for EPA 3.1 (below 0.7) in relation to both the students’ and supervisors’ ratings. - Students rated their ability to perform a task themselves higher in EPA 1.1 (p =0.006; d=0.377), EPA 2.9 (p =0.003; d=− 0.494) and EPA 3.1 (p = 0.017; d = 0.274), while supervisors rated the students’ ability higher in EPA 5.1 (p = 0.001; d = − 0.401) and 5.2 (p < 0.001; d = − 0.532). - The students’ self-rated ability to perform a task does not correlate with the su- pervisors’ ratings. The ratings of two supervisors on one student’s ability to perform a task correlate significantly in8ofthe12EPAs. - Based on the supervisors’ ratings for the 12 EPAs, a mean of 85% of the students reached supervision level 4 or higher, and a mean of 64% reached supervision level 5 or higher |
| 36) Vu et al. (2019) / USA/ Expert consensus | Internal medicine subinternship (four weeks duration) | The New Internal Medicine Subinternship Curriculum Guide: a Report from the Alliance for Academic Internal Medicine  Development   - Curriculum Objectives developed - EPAs mapped to milestones/competencies/RIME-Model - EPAs mapped to specialty specific competency framework | Curriculum based on 13 AAMC core EPAs:  1. Gather a history and perform a physical examination  2. Prioritize a differential diagnosis following a clinical encounter  3. Recommend and interpret common diagnostic and screening tests.  4. Enter and discuss orders and prescriptions.  5. Document a clinical encounter in the patient record.  6. Provide an oral presentation of a clinical encounter  7. Form clinical questions and retrieve evidence to advance patient care.  8. Give or receive a patient handover to transition care responsibility.  9. Collaborate as a member of an interprofessional team.  10. Recognize a patient requiring urgent or emergent care and initiate evaluation and management.  11. Obtain informed consent for tests and/or procedures.  12. Perform general procedures of a physician.  13. Identify system failures and contribute to a culture of safety and improvement.  Recommendations:   - Validated EPA-assessment tools needed - Distribute core EPA assessments over clerkships (e.g. EPA1, 2,3 4) and subinternships (e.g. EPA 10) |
| *AAMC: Association of American Medical Colleges  **UME: Undergraduate Medical Education, GME: Graduate Medical Education  ***AFMC: Association of Faculties of Medicine of Canada  ^Δ^ SMIFK/CIMS: Joint Commission of the Swiss Medical Schools  ψCodes adapted from O'Dowd, E., Lydon, S., O'Connor, P., Madden, C., & Byrne, D. (2019). A systematic review of 7 years of research on entrustable professional activities in graduate medical education, 2011–2018. Medical education, 53(3), 234-249. | | | |
